# Supplementary material for: Triboelectric Characterization of Colloidal TiO2 for Energy Harvesting Applications
Source: Nanomaterials (Basel). 2020 Jun 17;10(6):1181. doi: 10.3390/nano10061181 (PMC7353336; doi:10.3390/nano10061181)
Supplement: Supplementary file 1 [file nanomaterials-10-01181-s001.pdf]

nanomaterials

MDPI

SUPPORTING INFORMATION

Triboelectric Characterization of Colloidal TiO_2 for Energy Harvesting Applications

Erik Garofalo [1,2], Luca Cecchini [2], Matteo Bevione [1] and Alessandro Chiolerio [1,*]

1 Istituto Italiano di Tecnologia, Center for Sustainable Future Technologies, Via Livorno 60, 10144 Torino, Italy; erik.garofalo@iit.it (E.G.); matteo.bevione@iit.it (M.B.)
2 Politecnico di Torino, Department of Electronics and Telecommunications, Corso Duca degli Abruzzi 24, 10129 Torino, Italy; lucacecchini26@gmail.com
* Correspondence: alessandro.chiolerio@iit.it

Content: This file contains raw measurements used to extract data shown in Figure 6. For all materials investigated (DIW and TiO_2 suspensions at 0.5 and 1.0 wt.%), curves were taken at a fluid velocity of 3.4 cm/s.

Triboelectricity of DIW in a FEP pipe is shown in Figure S1.

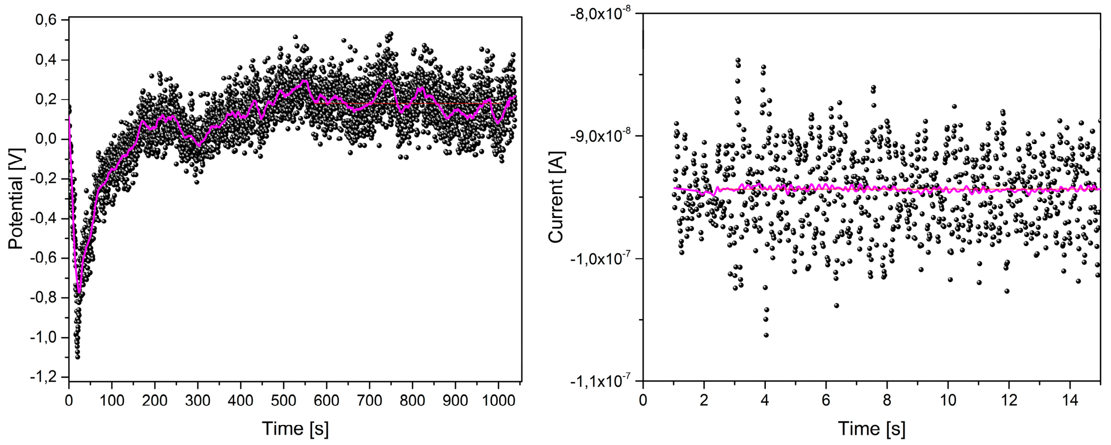

Figure S1. Open-circuit voltage (left) and short-circuit current (right) characterization of DIW.

Triboelectricity of 0.5 wt.% TiO_2 suspension in a FEP pipe is shown in Figure S2.

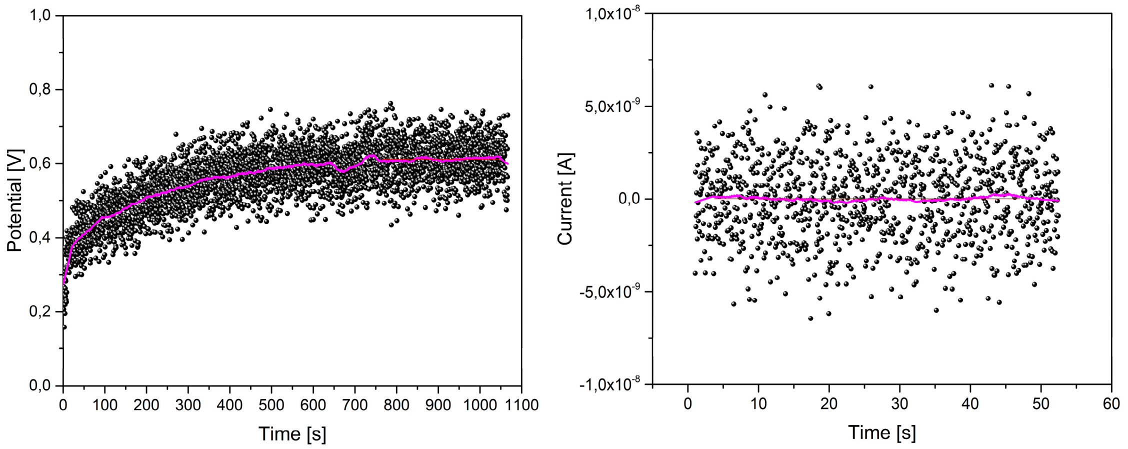

Figure S2. Open-circuit voltage (left) and short-circuit current (right) characterization of 0.5 wt.% TiO_2 suspension.

Triboelectricity of 1.0 wt.% TiO_2 suspension in a FEP pipe is shown in Figure S3.

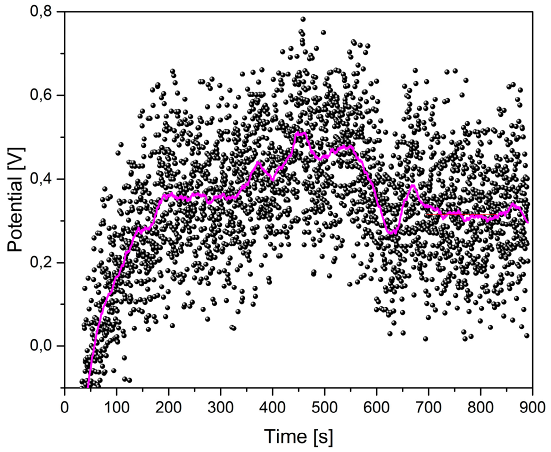

Figure S3. Open-circuit voltage (left) and short-circuit current (right) characterization of 1.0 wt.% TiO_2 suspension.

© 2020 by the authors. Submitted for possible open access publication under the terms and conditions of the Creative Commons Attribution (CC BY) license (http://creativecommons.org/licenses/by/4.0/).
